# Common Force Field Thermodynamics of Cholesterol

**DOI:** 10.1155/2013/207287

**Published:** 2013-11-05

**Authors:** Francesco Giangreco, Eiji Yamamoto, Yoshinori Hirano, Milan Hodoscek, Volker Knecht, Matteo di Giosia, Matteo Calvaresi, Francesco Zerbetto, Kenji Yasuoka, Tetsu Narumi, Masato Yasui, Siegfried Höfinger

**Affiliations:** ^1^Dipartimento di Chimica “G. Ciamician”, Università di Bologna, Via F. Selmi 2, 40126 Bologna, Italy; ^2^Department of Mechanical Engineering, Keio University, Yokohama 223-8522, Japan; ^3^Quantitative Biology Center, RIKEN Kobe Institute, Chuo-ku, Kobe 650-0047, Japan; ^4^Laboratory for Molecular Modeling and NMR Spectroscopy, National Institute of Chemistry, Hajdrihova 19, 1000 Ljubljana, Slovenia; ^5^Biomolecular Dynamics, Institute of Physics, Albert Ludwigs University, Hermann-Herder Straße 3, 79104 Freiburg, Germany; ^6^Faculty of Informatics and Engineering, University of Electro-Communications, 1-5-1 Chofugaoka, Chofu, Tokyo 182-8585, Japan; ^7^Keio School of Medicine, 35 Shinanomachi, Shinjuku-ku, Tokyo 160-8582, Japan; ^8^Department of Physics, Michigan Technological University, 1400 Townsend Drive, Houghton, MI 49931-1295, USA

## Abstract

Four different force fields are examined for dynamic characteristics using cholesterol as a case study. The extent to which various types of internal degrees of freedom become thermodynamically relevant is evaluated by means of principal component analysis. More complex degrees of freedom (angle bending, dihedral rotations) show a trend towards force field independence. Moreover, charge assignments for membrane-embedded compounds are revealed to be critical with significant impact on biological reasoning.

## 1. Introduction

Apart from a prominent role in cardiovascular disease [1, 2], cholesterol is also known for its largely varying membrane concentration in different tissues. For example, red blood cells exhibit concentrations of 50 mol%, significantly higher than the average 22 mol% in regular vertebrate cell membranes [3]. Even higher levels are seen in brain and nerve tissue, the metabolic significance of which is yet to be determined [4, 5]. Cholesterol is also an essential metabolic precursor for the biosynthesis of bile acids [6], steroid hormones [7], and vitamin D [8].

Owing to its importance, cholesterol has been the subject of intensive research over the last 70 years [9, 10]. Its complicated cellular location has led to a recent intensification of auxiliary methods with many of them entirely based on computer simulation. Indeed, many instructive examples have been given and the underlying computational models have advanced to a reasonable level of quality [11–17]. Such approaches are usually based on atomistic descriptions using empirical parameters to simulate the molecular mechanics/dynamics (MM/MD) of membrane lipids and their associated biomolecules. Commonly applied models are AMBER [18], CHARMM [19], GROMOS [20], OPLS [21], and the related simulation packages GROMACS [22], LAMMPS [23], NAMD [24], TINKER [25], and many more.

A general requirement for all force fields is that different parameter sets applied to the same molecule—cholesterol, for example—should yield comparable results and should not reveal significant differences. From the many comparisons available, those focussing on dynamic aspects appear to be particularly interesting [26–29]. Here we want to add to this type of dynamic assessment and present data for the thermodynamics of cholesterol using four different force fields from the above list. Applying* principal component analysis* (PCA) [30] we identify the most “influential” bonds/angles/dihedrals in a particular parameter set and compare them to each other, thus emphasizing the dynamic character of cholesterol when described by common force fields. 

## 2. Materials and Methods

### 2.1. AMBER(RESP)

A single cholesterol structure was model-built and optimized using Gaussian-09 [31] at the AM1 and B3LYP/3-21 G* level. The minimized geometry was considered at the HF/6-31 G* level and electrostatic potentials (ESPs) were computed for subsequent RESP assignment of atomic partial charges using ANTECHAMBER together with the GAFF force field (AMBER [18, 32] version 11). A single copy of RESP/GAFF-parameterized cholesterol was loaded into XLEAP and output in appropriate AMBER formats (prmtop/inpcrd), then minimized (2500 steps, cutoff 20 Å), and heated to 300 K target temperature (vacuum conditions) using Langevin dynamics, collision frequency *λ* = 1 ps^−1^, 12 Å cutoff, a time step of 1 fs, and no constraints on any bonds (i.e., SHAKE switched off). Identical conditions were applied during 5 ns of MD simulation where conformational snapshots were saved every 5000 steps to create a sample of 250 structures. 

### 2.2. AMBER(bcc)

Cholesterol was model-built and optimized at the HF/6-31 G** level using Gaussian-03 [31]. Atomic partial charges were assigned following the approach of AM1-bond charge correction (bcc) available in ANTECHAMBER of the AMBER package [18, 32] (version 8). The optimized structure was minimized (20 steps, steepest descent) and heated to the target temperature of 300 K within 100 ps of equilibration MD. Production MD over 5 ns used a time step of 1 fs, SHAKE constraints on XH bonds, a Berendsen thermostat, vacuum conditions without periodicity, and AMBER version 10. 

### 2.3. CHARMM

Cholesterol parameters were employed as reported previously [33]. A system containing a single copy of cholesterol was set up and heated to 300 K based on straight dynamics and CHARMM36 all-hydrogen lipid topology/CHARMM27 all-hydrogen lipid parameters [19]. Production MD was extended over a period of 5 ns using a time step of 1 fs, no SHAKE constraints, and the default cutoff of 12 Å specified in the cholesterol parameter file. 

### 2.4. GROMACS

Cholesterol parameters were used as reported previously [34]. A single molecule of cholesterol was put into a cubic box (58.219 Å^3^) and simulated at constant volume using periodic boundary conditions. All bonds were constrained [35], a time step of 2 fs was applied, total translation/rotation was periodically removed every 1000 steps, neighbour lists were updated every 5 steps, a cutoff of 10 Å was used, and the system was maintained at 300 K target temperature by means of a Nose-Hoover thermostat. 

### 2.5. PCA

All 250 snapshots collected by all the 4 different MD simulations were automatically converted from pdb format to xyz format and analyzed frame by frame for geometrical relationships (bonds/angles/dihedrals) with the help of TINKER [25] (module ANALYZE, option d) using the MM3 force field [36]. However, the MM3 force field was only a technical requirement for monitoring the actual dimensions of bonds/angles/dihedrals of a particular frame; hence these parameters were never really used or did never influence or alter any of the original geometries obtained by the 4 different force fields examined. PCA was then performed following the standard protocol [30]. A stand-alone solution was implemented in ANSI C supported by LAPACK [37]. 

### 2.6. ESPs (Membrane Internal)

**Figure 1:**
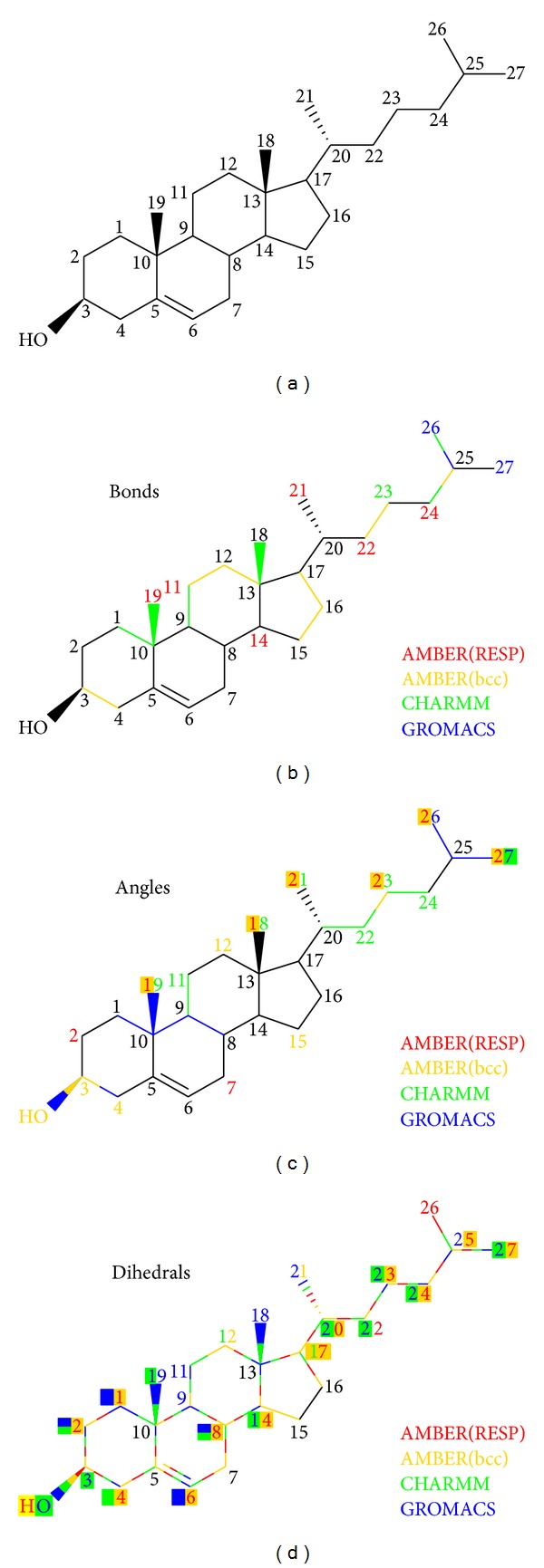
Principal component analysis (PCA) regarding 250 structural snapshots of cholesterol sampled over 5 ns of molecular dynamics (MD) simulations using common force fields. (a) Nomenclature of 3*β*-hydroxy cholesterol (numbering restricted to carbon atoms). (b) PCA of bonds (from a total of 77). (c) PCA of angles (157 angles in total). (d) PCA of dihedrals (259 dihedrals all in all). Color-coded are the top-ranked 10% bonds/angles/dihedrals based on a selection of PCs recovering 90% of the original data set. Different colors represent different force fields and involve C–H bonds if applied to numbers.

Mean structures were computed via superposition of all the 250 snapshots of all the 4 force fields on arbitrarily chosen reference frames. Mass-weighted all-atom fitting including H-atoms was performed using TINKER [25] (module SUPERPOSE) based on dummy employment of the MM3 force field [36] (see note in the previous section). The “most representative” structure for each of the 4 force fields was then determined as that frame that showed minimum RMSD from the average structure. A continuum description of the cellular membrane containing cholesterol was employed following the experimental findings of Ashcroft, Subczynski, and coworkers [38, 39]. Thus the OH-group and a small number of adjacent atoms on the cholesterol ring system (positions 2, 3, and 4 in Figure 1(a)) were assigned to the polar head group domain of the membrane which was represented by methanol [38]. The remaining part of cholesterol was assigned to the hydrophobic core domain of the membrane which was represented by cyclohexane [38]. Program POLCH [40] was used to compute ESPs following previous reports [15, 16, 41]. 

## 3. Results and Discussion

The standard nomenclature is adopted as schematically illustrated in Figure 1(a). Numbers are assigned to C-atoms only; hence missing H-atoms need to be considered whenever implicated in any of the identified bonds/angles/dihedrals. We started out to construct 4 different data sets of cholesterol conformations composed of 250 snapshots obtained from 5 ns of MD simulation based on AMBER(RESP) [18], AMBER(bcc) [32], CHARMM [19], and GROMACS [22]. The initial two descriptions differ with respect to the charge model applied. Either bonds (77 in total), angles (157), or dihedrals (259) were extracted from each of these 250 structures and written into separate data matrices which then became subject to PCA [30]. The top-ranked principal components, that is, specific linear combinations of the 77 bonds (or the other variables examined), will then identify those bonds (or the other variables examined), that experience the largest fluctuations, and hence are most relevant to the thermodynamics. Due to only marginal separation of eigenvalues corresponding to the top-ranked PCs, we took into account a subset, *j*, of PCs capable of reestablishing 90% of the original data set. We then isolated the top 10% components as follows,

(1) $$\begin{array}{c} x_{i}\;\;\forall i:i\in {\left\{\underset{j}{\sum }\lambda_{j}\left|c_{ij}\right|\right\}}_{{\text{TOP}}_{10\%}}, \end{array}$$

where *x*_*i*_ refers to a particular bond/angle/dihedral, *i*, part of the top 10% ranked coefficients, *c*_*ij*_, of the *j* PCs selected and weighted by their corresponding eigenvalue, *λ*_*j*_. PCA results for bonds are shown in Figure 1(b) where different colours symbolize different force fields. Independence of parameter sets is seen when a particular bond is identified several times; for example, the bond between C_25_ and C_26_ was top-ranked both by CHARMM and GROMACS (hence its colour of half blue, half green). The model most prone to bond variations was AMBER(bcc) involving exclusively C–C bonds (see yellow substructure in Figure 1(b)). In contrast, only C–H bonds were ranked by AMBER(RESP) (see red numbers in Figure 1(b)). However, this just reflects the employment/avoidance of SHAKE [42] constraints (see computational methods). CHARMM did identify a small set of both types, while GROMACS revealed only a minor group of bonds at the terminal end of cholesterol, a consequence of the restraints on all bonds [35] imposed during MD. In general, most of the bonds forming the ring system were not implicated in any of the top lists, an indication that the tetracyclic ring system maintains a rather rigid structure.

Next we turned our attention to the PCA of angles, and corresponding results are summarized in Figure 1(c). Again, the ring system was characterized as rather rigid, and many thermodynamically relevant angles did involve H-C-H groups with even exclusive preference by AMBER(RESP) (see red numbers in Figure 1(c)). Several hot spots of angle variation were identified to be located at terminal CH_3_-groups (see, e.g., C_19_ and C_27_). The overall impression gained was that there is a trend towards force field independence with increasing complexity of the type of motion analyzed. For example, the number of multiply identified sites of top-ranked angles did increase considerably when compared to the number of top-ranked bonds indicated by more than one force field. Even more impressive in this respect was the PCA of dihedrals (Figure 1(d)). Virtually all sites were marked by all force fields with the exception of only isolated positions inside the ring system.

**Figure 2:**
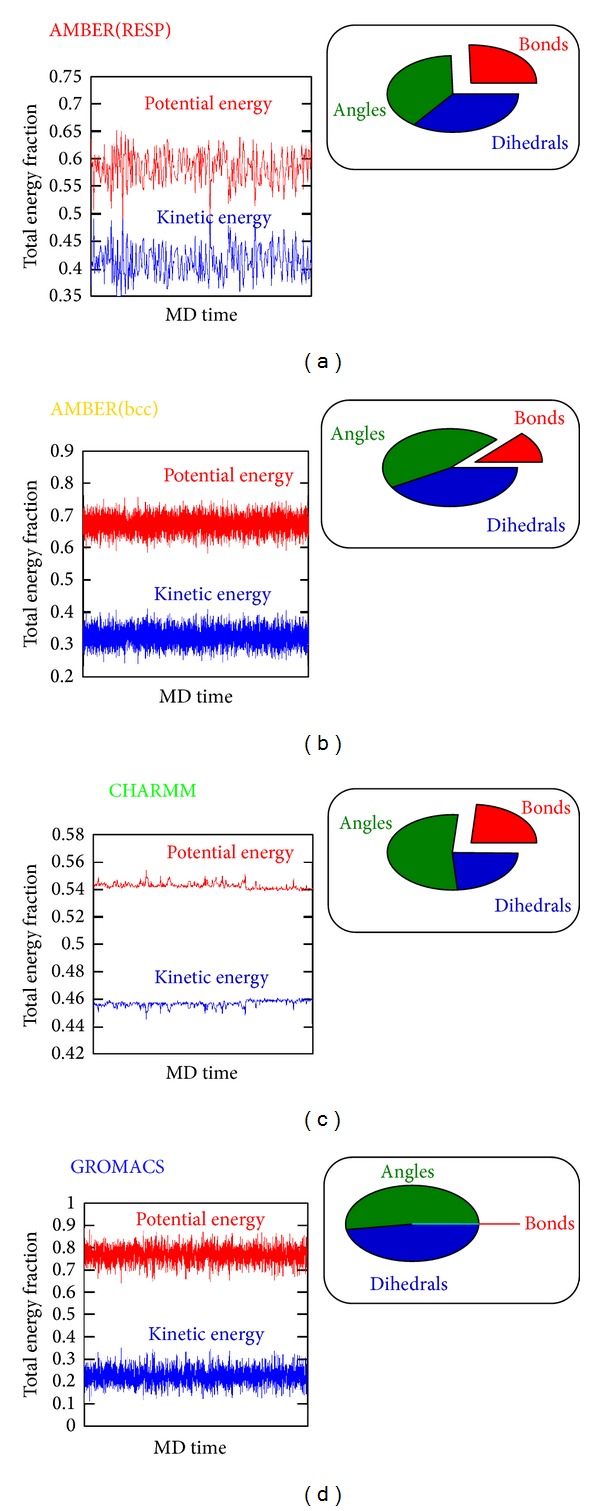
Relative contribution to the total energy from 5 ns of MD simulation (gas phase at 300 K) based on force fields: (a) AMBER(RESP), (b) AMBER(bcc), (c) CHARMM, and (d) GROMACS. All panels show the relative contribution of kinetic (blue) versus potential energy (red) to the total energy and the relative fraction of different types of potentials to the nonbonded energy (pie charts examining those degrees of freedom that have been characterized by PCA; see Figure 1).

In an attempt to link up our PCA data with MM energies we next determined trends of kinetic and potential energies and relative contributions to the nonbonded energy of the three types of motion studied. Results are summarized in Figure 2. The first interesting finding was that all the 4 force fields delivered net potential energies of positive sign but to a different relative extent. The two AMBER descriptions were roughly comparable, while CHARMM tended to an equipartition of energy between kinetic and potential forms, and GROMACS put more emphasis on potential energy (see relative contributions of red and blue graphs in Figures 2(a)–2(d)). In addition, significant differences were seen with respect to the degrees of fluctuations affecting kinetic and potential energies. Here, CHARMM revealed the trajectory progressing most smoothly, while AMBER and GROMACS exhibited comparable levels of “thermal noise.” Focussing on the relative contribution of the three types of motion analyzed by PCA, an approximately equal role of angles and dihedrals was observed for GROMACS, AMBER(RESP), and AMBER(bcc), while CHARMM prioritized contributions due to angles (compare sizes of blue and green areas of the pie charts in Figures 2(a)–2(d)). This may reflect the extra Urey-Bradley term of the CHARMM force field that was included in the contribution of angles when forming averages (green sector in Figure 2(c)). Relative contributions resulting from bonds (red sectors in the pie charts of Figure 2) turned out to be comparable between CHARMM and AMBER(RESP) but were less significant in AMBER(bcc) and entirely absent in GROMACS. The latter effect is the consequence of putting restraints on all types of bonds (GROMACS default mode [35]), not just on C–H bonds as commonly done by the other force fields.

**Figure 3:**
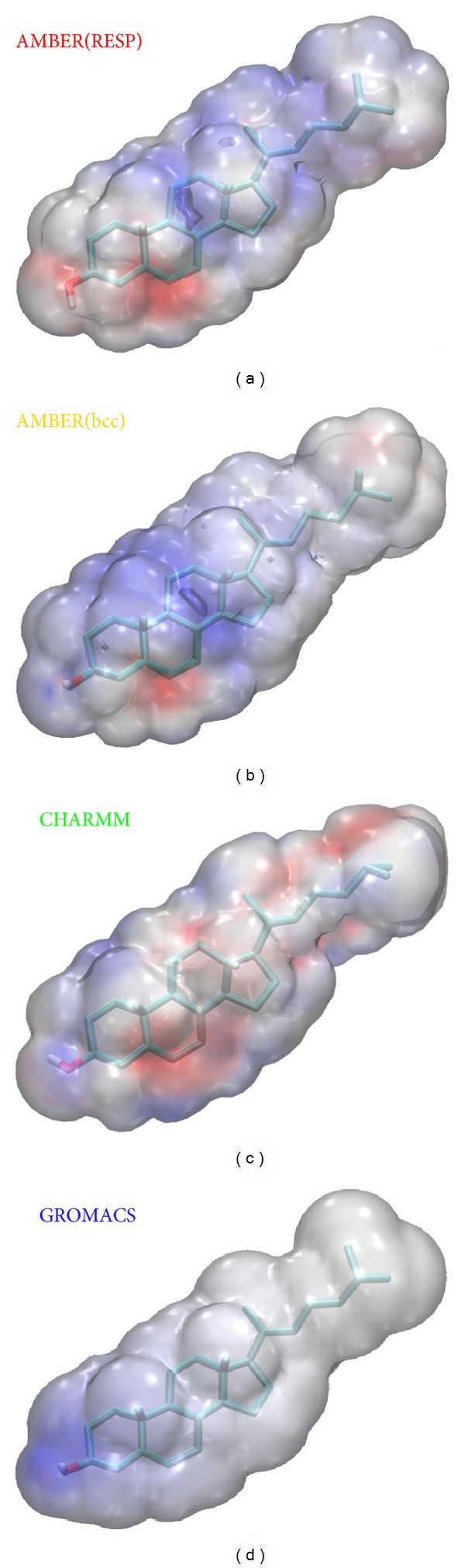
Membrane specific electrostatic potentials [15, 41] (ESPs) on the molecular surface of cholesterol. The assignment of partial charges is based on force fields: (a) AMBER(RESP), (b) AMBER(bcc), (c) CHARMM, and (d) GROMACS. Shown are color-coded ESPs (dark red: −5 *k*_*B*_ *T*/*q*_el_; dark blue: +5* k*_*B*_ *T*/*q*_el_) mapped onto the molecular surface of the average structure revealed from 5 ns of MD simulation. Standard or slightly increased van der Waals radii form the basis of molecular surface calculation.

Given the rather pronounced differences between AMBER(RESP) and AMBER(bcc) it appeared interesting to also examine different charge assignments and their corresponding electrostatic potentials (ESPs). Because we are interested in the effect of cholesterol on structure and function of cellular membranes, we applied a corresponding continuum description of such an environment [38, 39]. In practice, this means that only the OH-group and a small number of adjacent atoms of the cholesterol ring system (positions 2, 3, and 4 in Figure 1(a)) become exposed to the domain of polar head groups of the cellular membrane and thus will be assigned a medium of higher dielectric constant (i.e., comparable to methanol [38]), while the rest of the cholesterol structure will face the hydrophobic core environment of the membrane interior (modelled by cyclohexane) [15, 38]. We computed mean structures from the 250 explicit snapshots for all the 4 force fields tested and picked those snapshots that came closest to the average structure. Membrane specific ESPs were then computed based on the continuum description outlined above using an efficient computer model [40] that had recently been applied in a number of related studies of membrane associated biochemistry [15–17, 41]. Results are summarized in Figure 3 and visualized in Supporting Movie SI-CholESPs.mpg available online at http://dx.doi.org/10.1155/2013/207287. Main differences between CHARMM and AMBER (both models) became visible in the form of sizeable areas of positive ESPs displayed by the latter (see blue patches in both AMBER models of Figure 3 and Supporting Movie SI-CholESPs.mpg). AMBER(bcc) showed greater tendency to form such patches of positive ESPs when compared to AMBER(RESP). GROMACS on the other hand did not develop significant areas of nonneutral ESPs except for a single spot of positive ESPs in the vicinity of the OH-group. This is the natural consequence of a very restricted charge assignment by GROMACS involving only the OH-group and the anchor C-atom of cholesterol (position 3 in Figure 1(a)). Such marked differences in membrane internal ESPs may gain significant importance in properly explaining basic modes of receptor activation and signal transduction [43]. 

## 4. Conclusions

 In conclusion, the comparison of common force fields described here reveals a largely unifying picture of the structural dynamics of cholesterol and an increasing tendency of force field independence with more complex degrees of freedom such as angle bending and dihedral rotations (Figure 1). The methodic focus on dynamic aspects highlights the usefulness of nonenergy based techniques like, for example, PCA [30]. Our results clearly demonstrate that such a thermodynamic similarity is far from being obvious when strictly taking into account only partial contributions of kinetic and potential energies (Figure 2). In addition, we point out that particular care must be taken of realistic charge assignments for membrane-embedded compounds (Figure 3) since the effect on biomolecular interactions may be profound and consequences on biological reasoning may be severe [43].

## Supplementary Material

Supporting movie: represents an all-round view of membrane specific electrostatic potentials (ESPs, dark red: -5 kT/qel, dark blue: +5 kT/qel) computed on the molecular surface of cholesterol. Atomic partial charges of commonly applied biomolecular force fields form the basis of these ESPs.
